# Clinical Significance of a CD3/CD8-Based Immunoscore in Neuroblastoma Patients Using Digital Pathology

**DOI:** 10.3389/fimmu.2022.878457

**Published:** 2022-05-10

**Authors:** Liang Zeng, Shu-Hua Li, Shuo-Yu Xu, Kai Chen, Liang-Jun Qin, Xiao-Yun Liu, Fang Wang, Sha Fu, Ling Deng, Feng-Hua Wang, Lei Miao, Le Li, Na Liu, Ran Wang, Hai-Yun Wang

**Affiliations:** ^1^Department of Pathology, Guangzhou Women and Children’s Medical Center, Guangzhou Medical University, National Children’s Medical Center for South Central Region, Guangzhou, China; ^2^Molecular Diagnosis and Gene Testing Center, The First Affiliated Hospital of Sun Yat-Sen University, Guangzhou, China; ^3^Bio-totem Pte. Ltd., Foshan, China; ^4^Department of General Surgery, Nanfang Hospital, Southern Medical University, Guangzhou, China; ^5^Department of Molecular Diagnostics, Sun Yat-Sen University Cancer Center, State Key Laboratory of Oncology in South China, Collaborative Innovation Center for Cancer Medicine, Guangzhou, China; ^6^Guangdong Provincial Key Laboratory of Malignant Tumor Epigenetics and Gene Regulation, Cellular & Molecular Diagnostics Center, Sun Yat-Sen Memorial Hospital, Sun Yat-Sen University, Guangzhou, China; ^7^Departments of Thoracic Surgery, Guangzhou Women and Children’s Medical Center, Guangzhou Medical University, National Children’s Medical Center for South Central Region, Guangzhou, China; ^8^Department of Pediatric Surgery, Guangzhou Institute of Pediatrics, Guangdong Provincial Key Laboratory of Research in Structural Birth Defect Disease, Guangzhou Women and Children’s Medical Center, Guangzhou Medical University, National Children’s Medical Center for South Central Region, Guangzhou, China; ^9^Department of Experimental Research, State Key Laboratory of Oncology in Southern China, Collaborative Innovation Center for Cancer Medicine, Guangdong Key Laboratory of Nasopharyngeal Carcinoma Diagnosis and Therapy, Sun Yat-Sen University Cancer Center, Guangzhou, China; ^10^Department of Pathology, The First Affiliated Hospital of Sun Yat-Sen University, Guangzhou, China

**Keywords:** neuroblastoma, prognosis, immunology, digital pathology, CD3/CD8 T cells

## Abstract

**Background:**

Infiltrating immune cells have been reported as prognostic markers in many cancer types. We aimed to evaluate the prognostic role of tumor-infiltrating lymphocytes, namely CD3+ T cells, CD8+ cytotoxic T cells and memory T cells (CD45RO+), in neuroblastoma.

**Patients and Methods:**

Immunohistochemistry was used to determine the expression of CD3, CD8 and CD45RO in the tumor samples of 244 neuroblastoma patients. We then used digital pathology to calculate the densities of these markers and derived an immunoscore based on such densities.

**Results:**

Densities of CD3+ and CD8+ T cells in tumor were positively associated with the overall survival (OS) and event-free survival (EFS), whereas density of CD45RO+ T cells in tumor was negatively associated with OS but not EFS. An immunoscore with low density of CD3 and CD8 (CD3-CD8-) was indictive of a greater risk of death (hazard ratio 6.39, 95% confidence interval 3.09-13.20) and any event (i.e., relapse at any site, progressive disease, second malignancy, or death) (hazard ratio 4.65, 95% confidence interval 2.73-7.93). Multivariable analysis revealed that the CD3-CD8- immunoscore was an independent prognostic indicator for OS, even after adjusting for other known prognostic indicators.

**Conclusions:**

The new immunoscore based on digital pathology evaluated densities of tumor-infiltrating CD3+ and CD8+ T cells contributes to the prediction of prognosis in neuroblastoma patients.

## Background

Emerging evidence has demonstrated that tumor microenvironment (TME) could modulate cancer progression whereas the characteristics of TME may be used for molecular classification to predict treatment response and cancer survival (1, 2). Among different cell types observed in TME, tumor-infiltrating lymphocytes (TILs), including macrophages, dendritic cells, mast cells, natural killer cells, naive and memory lymphocytes, B cells, and effector T cells, are suggested as the main players in modulating cancer progression (3–5). Recent studies have indeed shown an association of TILs with the prognosis of several cancer types (5–7). For instance, a high density of infiltrating CD3+ T cells, CD8+ cytotoxic T cells and CD45RO+ memory T cells was reported to indicate a long overall survival (OS) and progression-free survival among patients with gastric, liver, lung, nasopharyngeal, and colorectal cancers (1, 8–11). Contradicting findings do also exist. For instance, one study reported that high density of CD8+ cytotoxic T cells was associated with larger tumor size in glioblastoma patients (12). Regardless, the existing literature does seem to suggest that TILs might have a generic role in the progression of many solid tumors.

Neuroblastoma (NB) is a common tumor deriving from sympathoadrenal progenitor cells within the neural crest and has substantially varying clinical courses ranging from spontaneous regression to widespread metastasis despite intensive therapy (13, 14). The International Neuroblastoma Staging System (INSS) is currently used to determine treatment strategy and assessment of NB prognosis. NB patients are also commonly classified as at high-, intermediate-, or low- risk of death according to the Children’s Oncology Group (COG) risk classification (15). The different risk groups might demonstrate specific genetic features. *TERT* rearrangement and inactivating mutations in *ATRX* have for instance been found predominantly among patients with high-risk NB (16). However, neither the staging systems nor the genetic variations seem to explain completely the extremely divergent clinical course of NB.

With the wide application of cancer immunotherapies, it becomes increasingly important to understand the immunological landscape of the TME to understand the divergent course of NB and to predict treatment response. A recent study showed that CD4+ T cells and macrophages are involved in the oncogenesis of NB (17). Marco et al. showed that tumor-infiltrating T cells, mainly CD3+, CD4+, and CD8+ T cells, improved clinical outcomes of patients with therapy-resistant NB (18) whereas Riyue et al. demonstrated that patients with high-risk NB showed a better survival when the tumor was T cell inflamed (19). A detailed description of the phenotypes of all infiltrating T cells, especially CD3+ T cells, CD8+ cytotoxic T cells, and CD45RO+ memory T cells, using a large collection of NB specimens is however currently not available.

To this end, we investigated the densities of tumor-infiltrating CD3+, CD8+ and CD45RO+ T cells in a large sample of NB patients using digital pathology and developed a new immunoscore, summarizing such densities, to assess its role in predicting OS and event-free survival (EFS) of NB patients.

## Patients and Methods

### Clinical Specimens

**Figure 1:**
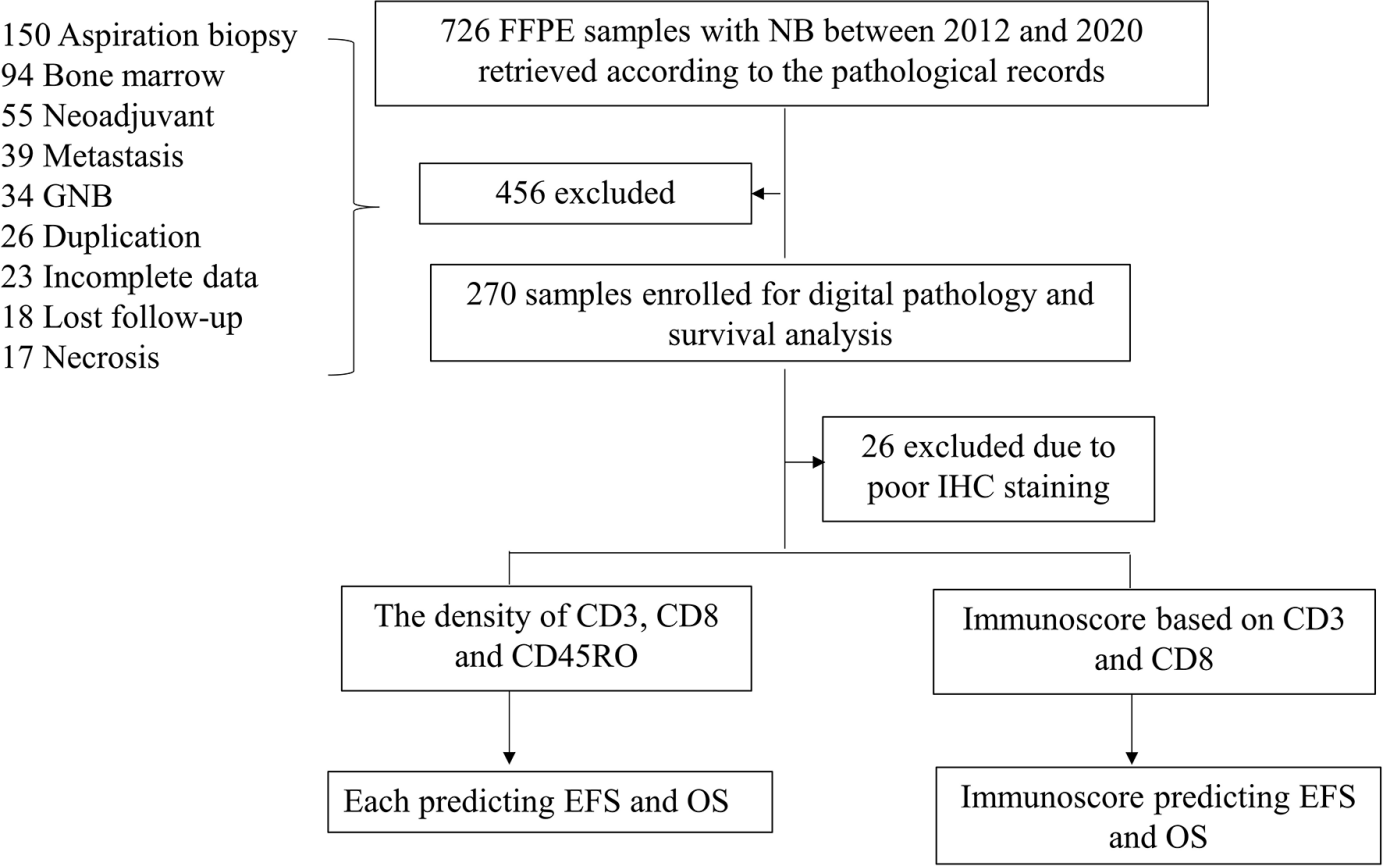
Workflow of the present study. Abbreviations: GNB, ganglioneuroblastoma; NB, neuroblastoma; IHC, immunohistochemistry; EFS, event-free survival; OS, overall survival.

All tumor specimens of 726 NB patients collected between January 2012 and December 2020 were identified in two academic institutions, the Guangzhou Women and Children’s Medical Center (GWCMC) and the First Affiliated Hospital of Sun Yat-sen University (SYSU), in Guangdong Province, China. Among these, we included 244 NB patients with a newly diagnosed NB during this period and at an age <15 years (**Figure 1**). Only specimens with a tumor content greater than 50% were included in the study. All diagnoses were pathologically confirmed and restaged according to the INSS (20). The COG risk classification was performed for each patient according to medical records. This study is reported according to the Reporting Recommendations for Tumor Marker Prognostic Studies criteria (21). An Institutional Review Board approved the ethical, legal, and social implications of this project. Parents of all NB patients provided written informed consent to the participation of the study, mostly at the time of admission to the two academic institutions.

### Immunohistochemical Staining

Pathological sequential slides of 4 µm thickness were sliced up from the formalin-fixed, paraffin-embedded (FFPE) tumor blocks and used for immunohistochemistry (IHC) analysis. Primary antibodies used were CD3 (Catalog #ab16669, 1:800; Abcam, Cambridge, UK), CD8 (Catalog #ab4055, 1:800; Abcam, Cambridge, UK), and CD45RO (clone UCHL1, 1:1600; Cell Signaling Technology, CST, Beverly, Massachusetts, USA). IHC was conducted as previously described (22). **Supplementary Figure 1** shows example images of IHC staining with high, low, and negative expressions of all three markers (online only).

### Digital Pathology

**Figure 2:**
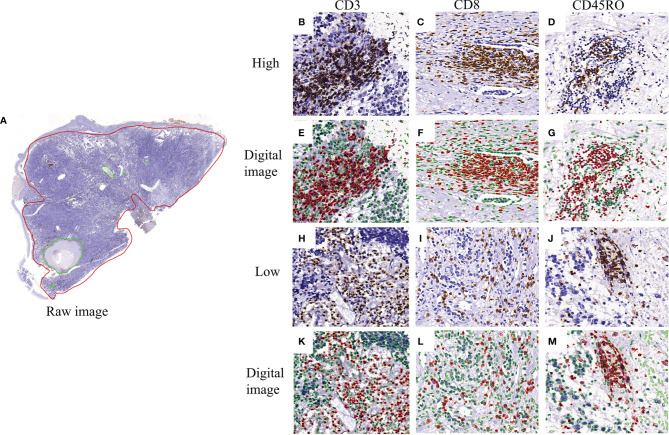
Representative image of CD3+, CD8+, and CD45RO+ expression and digital pathology. **(A)** a full view of immunohistochemistry staining labeled by the red circle as the tissue areas including tumorous and stromal areas and green circle as the necrosis areas; **(B–D)** the high expression of CD3+, CD8+ and CD45RO+ T cells; **(E–G)** the corresponding visual nucleus segmentation images in red color as positive and in green color as negative; **(H–J)** the low expression of CD3+, CD8+ and CD45RO+ T cells; **(K–M)** the corresponding visual nucleus segmentation images in red color as positive and in green color as negative.

Physical glass slides stained with CD3, CD8, and CD45RO were first reviewed by two experienced pathologists (L.Z and K.C) to ensure good staining quality for downstream analysis. All slides were then digitized at x200 magnification (Pannaromic Scan 150, 3DHistech, Hungary). Tumor areas with tumor nests and surrounding stroma areas, as well as necrosis areas, were manually annotated by a pathologist (K.C), using the QuPath software (version 0.2.3, University of Edinburgh, Scotland) (**Figure 2A**). Regions-of-interest (ROI) was defined as annotated tumor areas excluding necrosis areas. The density of positively stained cells in the ROIs was quantified in the following steps. Stain deconvolution (23) was first performed to separate the original images into haematoxylin channel and DAB channel images. Nuclei were segmented from the haematoxylin channel images using a Mask-RCNN based deep learning method as described in a previous study (24). The positively stained areas were identified from the DAB channel images using a pre-defined cutoff value obtained according to the control slides. A positive cell was identified if the positively stained areas were found to overlap with the segmented nucleus region for CD3 and CD8 stained images or with the ring-shape surrounding nucleus region for CD45RO stained images. Finally, the numbers of CD3+, CD8+ and CD45RO+ T cells were quantified from the ROIs of each image and recorded as density per mm^2^. **Figures 2B**–**M** show example images with high and low numbers of CD3+, CD8+, and CD45RO+ T cells.

### Cut-Off Values for High or Low Densities

We used the ‘Survminer’ package in R software (version 4.1.2) to calculate optimal cut-off values to define a high or low density of CD3+, CD8+ and CD45RO+ T cells, using OS as the outcome. Based on such cutoff values, each patient was given a binary score, 0 for low density indicated as (-) and 1 for high density indicated as (+), for each cell type (CD3+, CD8+ and CD45RO+).

### Statistical Analysis

Descriptive statistics were provided for clinical characteristics of the NB patients. The primary outcome was OS and secondary outcome was EFS, as identified through medical records. OS was calculated from the date of cancer diagnosis to the date of death from any cause. EFS was determined from the date of cancer diagnosis to the first occurrence of any event (i.e., relapse at any site, progressive disease, second malignancy, or death). Patients without an event were censored on the date of last contact (25).

The correlations between the expression levels of CD3, CD8, and CD45RO and NB clinical variables were analyzed using the χ2 test or Fisher’s exact test. We used Kaplan-Meier curves with the log-rank test method to estimate the differences in OS and EFS between patients with high and low densities of CD3+, CD8+ or CD45RO+ T cells. We also compared patients with different value of an immunoscore created based upon these individual markers. The decision of creating an immunoscore was made *a priori* given previous studies of similar kind using digital pathology (11). Hazard ratios (HRs) with 95% confidence intervals (CI) were first calculated using a univariate Cox regression analysis to show the associations of different prognostic indicators, including the densities of CD3+, CD8+, and CD45RO+ T cells and the combined immunoscore, with the risk of death or any event. Multivariable Cox regression analysis with backward selection was then used to test the prognostic roles of these different factors, mutually adjusted for one another. Only factors showing a statistically significant association (P < 0.05) in the univariable analysis were included in the multivariable analysis. We analyzed CD3+, CD8+, and CD45RO+ T cells separately in the first multivariable model and as a combined immunoscore in the second multivariable model.

Statistics analyses were performed using R (version 4.1.2) and Stata version 15.1 (Texas, USA). All statistical tests were two sided and considered significant when the P value was less than 0.05.

## Results

### Patient Characteristics and the Expression of CD3, CD8 and CD45RO

**Table 1** displays the patient characteristics. We evaluated the numbers of infiltrating total T cells (CD3+), cytotoxic T cells (CD8+), and memory T cells (CD45RO+) per mm^2^ in the NB tissue blocks. A wide spectrum of infiltrating immune cells in the tissue blocks were identified using CD3, CD8, and CD45RO antibodies. The highest density was found for CD3 (median: 156, range: 0-2100/mm^2^) followed by CD8 (median: 90, range: 0-864/mm^2^) and CD45RO (median: 14, range: 0-949/mm^2^) (P<0.001; **Supplementary Figure 2A**). The densities by age, pathological grade, MYCN status, risk classification, INSS, and gender are shown in **Supplementary Figures 2B**–**G** (online only). The 3-year OS and EFS were 77.1% (70.8-82.3%) and 63.0% (56.2-69.1%), whereas the 5-year OS and EFS were 70.1% (62.4-76.6%) and 60.5% (53.2-66.9%) in the 244 NB patients.

**Table 1:** Clinicopathological characteristics of the 244 NB patients included in the study.

Characteristics	No. (%)
**Age, months**	
< 18	114 (46.7)
≥ 18	130 (53.3)
**Sex**	
Female	104 (42.6)
Male	140 (57.4)
**Pathological grade**	
Well differentiated	78 (32.0)
Undifferentiated or poorly differentiated	163 (66.8)
NA	3 (0.2)
**MKI**	
High	45 (18.4)
Intermediate	30 (12.3)
Low	155 (63.5)
NA	14 (5.8)
**MYCN status**	
Amplified	33 (13.5)
Nonamplified	190 (77.9)
NA	21 (8.6)
**INSS**	
Early stage	80 (32.8)
Advanced stage	164 (67.2)
**COG risk group**	
High	108 (44.3)
Intermediate	62 (25.4)
Low	74 (30.3)
**3-year OS (95% CI)**	77.1% (70.8%-82.3%)
**3-year EFS (95% CI)**	63.0% (56.2%-69.1%)
**5-year OS (95% CI)**	70.1% (62.4%-76.6%)
**5-year EFS (95% CI)**	60.5% (53.2%-66.9%)

NB, neuroblastoma; MKI, mitosis-karyorrhexis index; INSS, the International Neuroblastoma Staging System; COG, Children’s Oncology Group; OS, overall survival; EFS, event-free survival; CI, confidence intervals; NA, not applicable.

### Prognostic Values of Individual Markers

**Figure 3:**
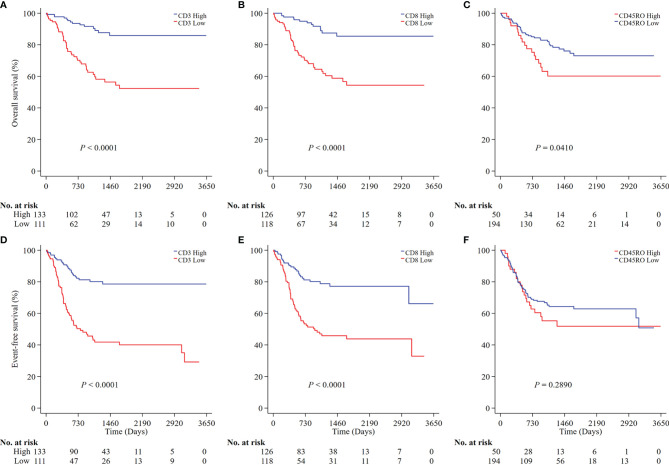
Survival analysis of CD3, CD8 and CD45RO expression in all NB patients. Kaplan-Meier survival curves were performed according to the high and low densities of CD3, CD8 and CD45RO (**A–C** for OS; **D–F** for EFS). P values were calculated by Log-rank test. NB, neuroblastoma; OS, overall survival; EFS, event-free survival.

We next analyzed the prognostic values of CD3, CD8, and CD45RO in NB. A low density of CD3+ T cells was associated with a lower OS (P<0.0001; **Figure 3A**) and a higher risk of death (HR 4.46, 95% CI 2.44-8.17) (**Supplementary Table 1**, online only). Similarly, a low density of CD8+ T cells was also associated with a lower OS (P<0.0001; **Figure 3B**) and a higher risk for death (HR 4.25, 95% CI 2.29-7.90) (**Supplementary Table 1**). Similar results were found for EFS (P<0.0001 for both CD3+ and CD8+ T cells; **Figures 3D, E**) and the risk of any event (HR 3.78, 95% CI 2.38-6.02 for CD3+ T cells and HR 3.06, 95% CI 1.94-4.84 for CD8+ T cells (**Supplementary Table 1**). A low density of CD45RO+ T cells was associated with a greater OS (P=0.0410; **Figure 3C**) and a lower risk of death (HR 0.55, 95% CI 0.31-0.95) (**Supplementary Table 1**) but not any event (**Figure 3F** and **Supplementary Table 1**).

### Prognostic Value of the Immunoscore

**Figure 4:**
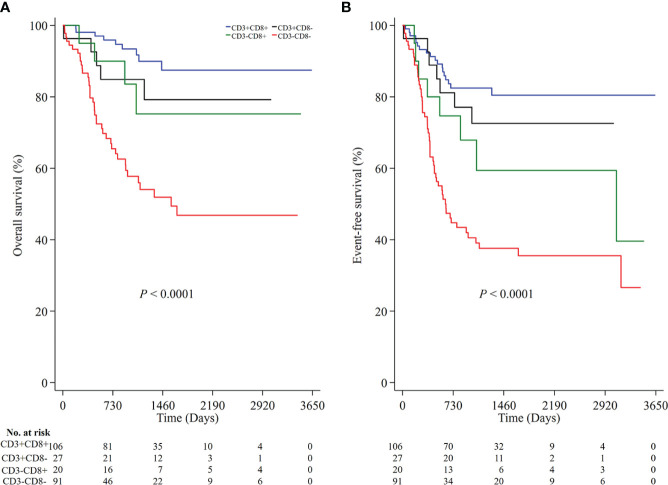
The prognostic value of the immunoscore based on the high and low densities of CD3 and CD8 in all NB patients. Kaplan-Meier survival analysis with the log-rank test was performed according to the immunoscore (**A**, P < 0.0001 for OS; **B**, P < 0.0001 for EFS). P values were calculated by Log-rank test. NB, neuroblastoma; OS, overall survival; EFS, event-free survival.

To understand the joint prognostic value of these immune markers, we developed an immunoscore (IS) summarizing the individual markers. As CD45RO+ T cells was not associated with EFS in the above analysis, we classified all NB patients into four groups according to the densities of CD3 and CD8, namely high densities of both CD3 and CD8 (CD3+CD8+), high density of CD3 but low density of CD8 (CD3+CD8-), low density of CD3 but high density of CD8 (CD3-CD8+) and low densities of both CD3 and CD8 (CD3-CD8-). The immunoscore of CD3-CD8- was associated with a lower OS (P<0.0001 for both OS and EFS; **Figures 4A, B**) and a higher risk of both death and any event (HR 6.39, 95% CI 3.09-13.20 for death and HR 4.65, 95% CI 2.73-7.93 for any event (**Supplementary Table 1**).

As age, MYCN status, INSS, and COG risk were statistically significantly associated with OS and EFS in the univariable analysis (**Supplementary Table 1**), we performed multivariate Cox regression analysis to assess the roles of the individually markers as well as the immunoscore after adjustment for age, MYCN status, INSS, and COG risk in two separate models. In the model for individual markers, we found similar associations between CD3+ and CD8+ T cells with the risk of death or any event, as in the univariable analysis (**Supplementary Table 2**, online only). In the model for immunoscore, we found that the immunoscore of CD3-CD8- was still associated with a higher risk of death and any event after adjustment for all other prognostic factors (HR 6.04, 95% CI 2.83-12.86, P < 0.0001 for death; HR 4.45, 95% CI 2.55-7.76, P<0.0001 for any event) (**Table 2**).

**Table 2:** Multivariable Cox regression analysis of risk factors associated with OS and EFS in NB patients.

Characteristics		Multivariable HR (95% CI)	*P* value		Multivariable HR (95% CI)	*P* value
	OS			EFS		
**Age, months**			0.02			0.83
< 18		Reference			Reference	
≥ 18		3.00 (1.17-7.70)			0.93 (0.48-1.80)	
**MYCN status**			0.18			0.89
Amplified		Reference			Reference	
Nonamplified		0.64 (0.33-1.22)			0.96 (0.54-1.69)	
**INSS**			0.60			0.58
Advanced stage		Reference			Reference	
Early stage		0.68 (0.17-2.78)			0.74 (0.27-2.08)	
**COG risk**						
High		Reference			Reference	
Intermediate		0.48 (0.17-1.37)	0.17		0.35 (0.16-0.75)	0.01
Low		0.14 (0.02-1.09)	0.06		0.09 (0.02-0.38)	0.00
**Immunoscore**						
CD3+CD8+		Reference			Reference	
CD3+CD8-		2.50 (0.82-7.63)	0.11		1.81 (0.74-4.38)	0.19
CD3-CD8+		1.07 (0.23-4.99)	0.93		1.96 (0.77-4.97)	0.16
CD3-CD8-		6.04 (2.83-12.86)	< 0.0001		4.45 (2.55-7.76)	< 0.0001

MKI, mitosis-karyorrhexis index; INSS, the International Neuroblastoma Staging System; COG, Children’s Oncology Group; OS, overall survival; EFS, event-free survival; HR, hazard ratio; CI, confidence interval; NA, not applicable; P < 0.05 indicates statistical significance.

## Discussion

In a study of 244 incident patients with NB, we performed the IHC analysis and digital pathology to assess the prognostic values of the densities of tumor-infiltrating CD3+, CD8+ and CD45RO+ T cells in NB survival. We found a high density of CD3+ and CD8+ T cells to indicate longer OS and EFS whereas an immunoscore of low densities of both CD3 and CD8 to indicate the highest risk of death or any progression event.

A previous study has reported that TME within tumor regions, including tumor interior and invasive margins, may be defined by the type, functional orientation, density, and location of immune cells (26). Therefore, tumor-infiltrating immune cells have obtained considerable research interest, especially in terms of their prognostic values in treatment response and caner survival (27). Several studies have shown that the density and distribution of TILs based on H&E slides are valuable prognostic makers in breast (28), non-small cell lung (29) and gastric (30) cancers. Moreover, studies have shown that the density of TILs is independently associated with treatment response of neoadjuvant chemotherapy in triple-negative breast cancer with HER2-overexpressing disease and homologous recombination deficient status (31, 32).

Galon et al. reported however that using H&E slides to evaluate density of TILs was not reproducible and highly subjective, and suggested that immunoscore through quantifying specific T cells such as CD3+, CD8+ and CD45RO+ T cells might be more reproducible and objective (1). Such immunoscore has indeed been reported as prognostic indicators in many cancers. Frank et al. showed for instance that CD3+, CD8+ and CD45RO+ T cells were correlated with cancer recurrence and OS in patients with rectal cancer and an immunoscore integrating the three was a powerful prognostic tool and might supplement the TNM staging system (33). In this study, we used digital pathology and showed that CD3+ T cells and CD8+ T cells mirrored each other as previously shown in non-small cell lung cancer and colorectal cancer (1, 10), similarly suggesting that high densities of CD3+ and CD8+ T cells were associated with a longer OS in NB.

High densities of TILs appear to decrease the risk of cancer recurrence, however, the driving forces for the differential densities remain unknown. It has been speculated that both host and tumor factors might contribute to the density of TILs. Therefore the densities of CD3+, CD8+, and CD45RO+ T cells in NB may also differ by clinical variables. In our study, we found that the density of CD3+ T cells was higher than that of CD8+ and CD45RO+ T cells. Further, density of CD3+ T cells was higher among NB patients with MYCN nonamplification and male, density of CD8+ T cells was higher among NB patients with differentiated pathology and male, whereas density of CD45RO+ T cells was higher among NB patients diagnosed above 18 months of age. These suggest that CD3+, CD8+ and CD45RO+ T cells might have different roles in the prognosis of NB, namely that CD3+ T cells are mostly strongly associated with OS and EFS of NB, followed by CD8+ T cells, as suggested in a previous study (18), whereas CD45RO+ T cells might have little or a harmful effect on NB survival. In general, a high density of CD45RO has been related to good clinical outcomes of several solid tumors, including melanoma, head and neck cancer, lung cancer, and colorectal cancer (34). The contradictory finding of NB and the previous studies might suggest that the role of CD45RO might be specific to tumor type and TME. Indeed, in contrast to other solid tumors, NB is a pediatric cancer with low mutation burden, little infiltrating immune cells, and poor response to immune checkpoint inhibitors (35, 36).

It is known that the tumor microenvironment is both diverse and complicated as different immune cells are capable of infiltrating tumor tissues, indicating that a synergistic view of different immune cells might provide better understanding of the immune microenvironment. For example, one study reported that the number of CD8+ TILs alone could not predict survival of patients with glioma, whereas the combination of having both low density of CD8+ and high density of CD4+ predicted survival (33). In the present study, we classified the NB patients into four groups based on the densities of CD3+ and CD8+ T cells and found that NB patients with CD3-CD8- had the highest risk of death and progression event, compared with other patients. Our results add therefore important new knowledge to existing literature suggesting a beneficial effect of cytotoxic T lymphocytes in tumors of diverse origin (11, 26, 31).

Digital pathology is gaining momentum in the analysis of pathologic tissue samples due to its accuracy and quantification of the full-view slides, providing a tool for more automatic and objective evaluation (37). Another advantage is the possibility to facilitate complex spatial patterns and standard metrics (38). In our study, we conducted digital pathology to evaluate the densities of CD3+, CD8+ and CD45RO+ T cells in NB tumor tissues. Due to the lower overall densities of infiltrating immune cells in NB tumors, compared to other cancers, we evaluated the densities of these markers in the indicated slides as a whole. Franck et al. have also used digital pathology and presented an immunoscore based on the densities of CD3+ and CD8+ T cells measured in the core and invasive margins of tumors, and found it to predict the survival of patients with rectal cancer (33). Therefore, immunoscore using digital pathology made it possible to determine the immunotype for each NB patient, strongly indicative of prognosis.

Novel tumor classification has also been proposed based on the immunoscore, as hot (high immunoscore), altered (intermediate immunoscore), and cold (low immunoscore) tumors (39). Immunotherapies are the most rapidly growing field in cancer treatment and have a major impact in clinical outcomes of cancer patients. A consensus was made that effector T cells play the central role in antitumor responses (40). Several studies have reported that hot tumors show a fertile ground for effective immune checkpoint inhibitors (ICIs) based on monotherapy or combination therapies, whereas cold tumors featured by low immunoscore should be treated with combined priming therapy, which may enhance T cell responses to convert an immune cold tumor to an immune hot tumor (41). NB is in general characterized as low immunogenicity due to the lower tumor mutation burden and lower infiltrating of immune cells (42), impeding the effective engagement during immunotherapy (43). Thus, a different or more extensive biomarker should be developed for successful implementation of novel immunotherapies in NB patients to improve survival.

There are several limitations in this study. First, we did not evaluate TILs density using H&E slides. However, it remains interesting to incorporate information on the presence of tertiary lymphoid structures (TLSs) and TILs to the immunoscore, which will likely lead to better understanding of the functional role of immune contexture in NB. Another limitation is the lack of independent validation. Future studies of independent samples are therefore needed to validate our findings.

In conclusion, our work shows that high densities of CD3+ and CD8+ T cells are indicative of longer OS and EFS in NB patients. Additionally, an immunoscore based on the densities of CD3+ and CD8+ T cells may help to classify patients with NB into different prognostic profiles. Together, these findings might pave the way for a novel implementation of immunotherapy in NB patients.

## Data Availability Statement

The raw data supporting the conclusions of this article will be made available by the authors, without undue reservation.

## Ethics Statement

The studies involving human participants were reviewed and approved by the Institute Research Ethics Committee of Guangzhou Women and Children’s Medical Center [2021]078A01. Written informed consent to participate in this study was provided by the participants’ legal guardian/next of kin.

## Author Contributions

H-YW and NL performed study concept and design. LZ, S-HL, KC, and L-JQ conducted experiment. LZ and KC reviewed IHC slides. S-YX conducted digital pathology. H-YW, LZ, X-YL, S-YX, and RW performed data analysis and interpretation. FW, SF, S-HL, F-HW, LL, and LM retrieved clinical data. H-YW, LZ, and X-YL wrote manuscript drafting. All authors have reviewed and approved the final manuscript for publication.

## Funding

This work was partially supported by the start-up program of Guangzhou Women and Children’s Medical Center (No. 3001151-04) and the National Natural Science Foundation of China (81602468).

## Conflict of Interest

Author S-YX was employed by Bio-totem Pte. Ltd.

The remaining authors declare that the research was conducted in the absence of any commercial or financial relationships that could be construed as a potential conflict of interest.

The reviewer SW declared a shared affiliation with the authors X-YL, FW, LD, and NL to the handling editor at the time of review.

## Publisher’s Note

All claims expressed in this article are solely those of the authors and do not necessarily represent those of their affiliated organizations, or those of the publisher, the editors and the reviewers. Any product that may be evaluated in this article, or claim that may be made by its manufacturer, is not guaranteed or endorsed by the publisher.

## Abbreviations

NB, Neuroblastoma; INRG, International Neuroblastoma Risk Group; TME, tumor microenvironment; INSS, International Neuroblastoma Staging System; IHC, immunohistochemistry; FFPE, formalin-fixed, paraffin-embedded; OS, overall survival; EFS, event-free survival; COG, Children’s Oncology Group.
