# Supplementary material for: Clinical Significance of a CD3/CD8-Based Immunoscore in Neuroblastoma Patients Using Digital Pathology
Source: Front Immunol. 2022 May 10;13:878457. doi: 10.3389/fimmu.2022.878457 (PMC9128405; doi:10.3389/fimmu.2022.878457)
Supplement: Supplementary file 1 [file DataSheet_1.docx]

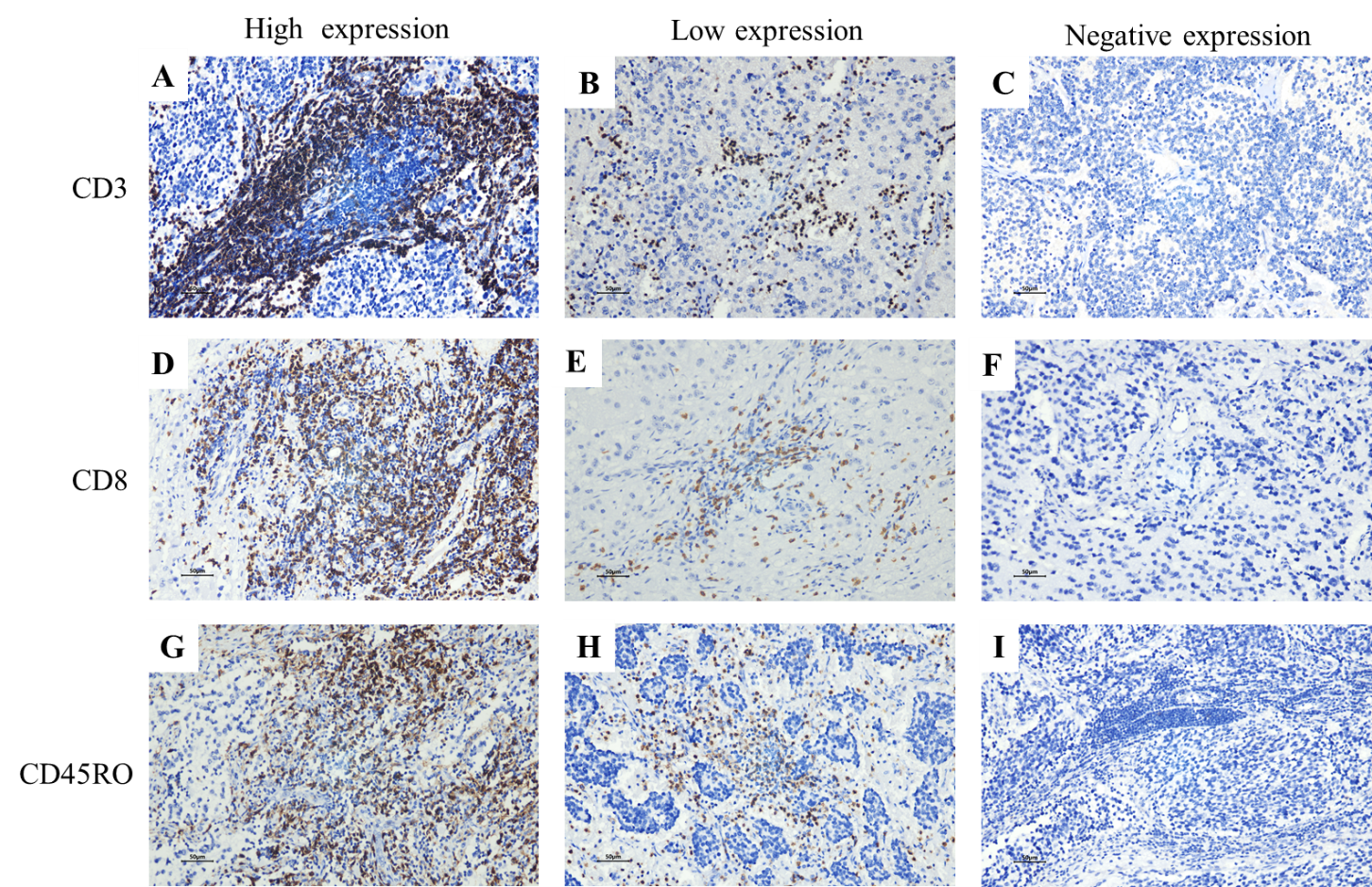


**Supplementary Figure S1.** Representative images of immunohistochemistry staining with CD3，CD8, and CD45RO markers in neuroblastoma samples. High expression of CD3, CD8, and CD45RO are shown in box A, D, and G. Low expression of CD3, CD8, and CD45RO are shown in box B, E, and H. Negative expression of CD3, CD8, and CD45RO are shown in box C, F, and I.
