# Supplementary material for: Clinical Significance of a CD3/CD8-Based Immunoscore in Neuroblastoma Patients Using Digital Pathology
Source: Front Immunol. 2022 May 10;13:878457. doi: 10.3389/fimmu.2022.878457 (PMC9128405; doi:10.3389/fimmu.2022.878457)
Supplement: Supplementary file 2 [file DataSheet_2.docx]

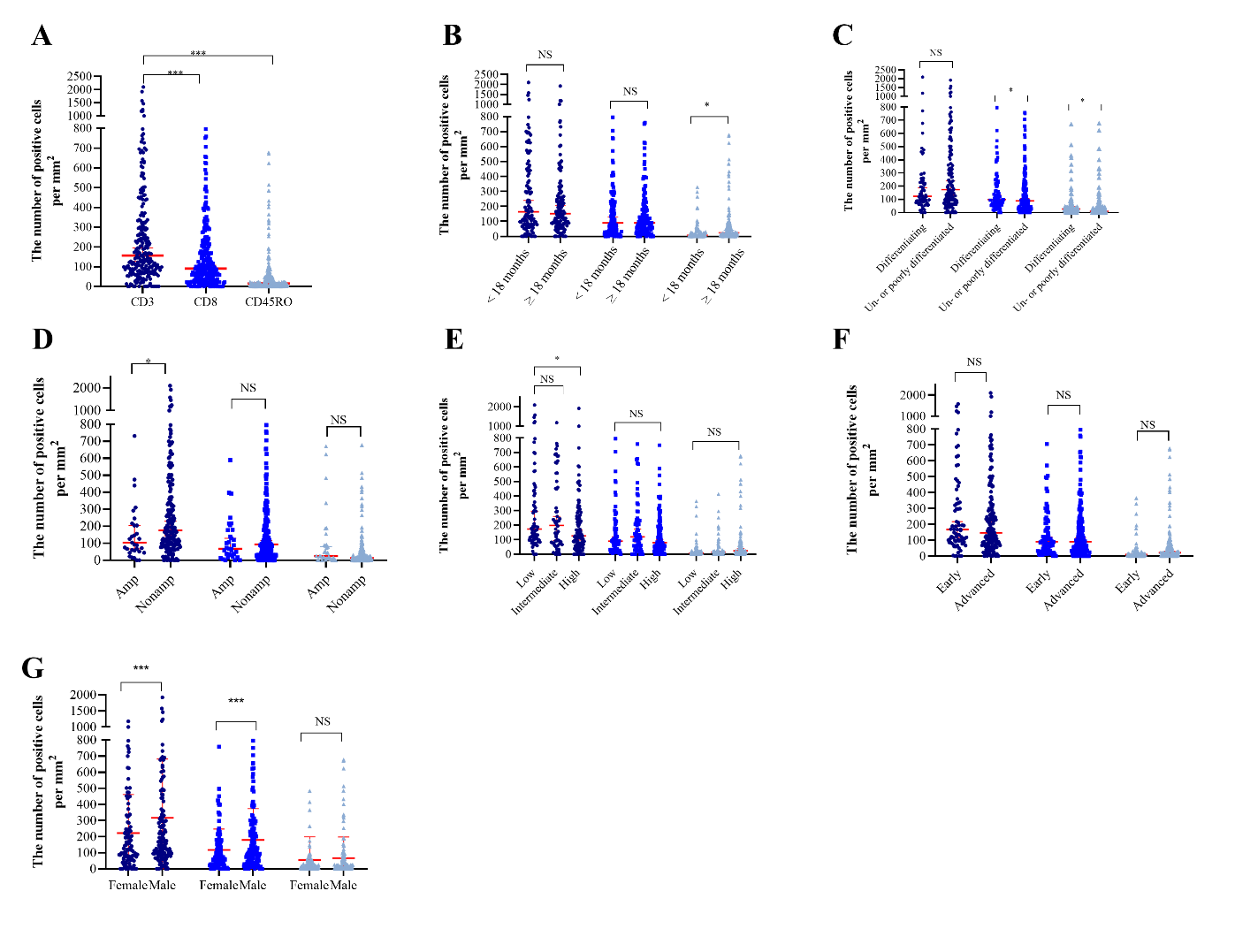


**Supplementary Figure S2.** Tumor-infiltrating densities of CD3 (dark blue), CD8 (blue), and CD45RO (light blue) in neuroblastoma patients. A: all patients. B: by age (<18 or >18 months). C: by tumor differentiation. D: by MYCN status. E: by Children’s Oncology Group risk classification. F: by International Neuroblastoma Staging System. G: by gender. *P<0.05, **P<0.01, ***P<0.001, NS, no significant.
